# Patient experiences of living with cancer before interaction with palliative care services in Zimbabwe: A qualitative secondary data analysis

**DOI:** 10.1111/ecc.13632

**Published:** 2022-06-17

**Authors:** Adlight Dandadzi, Emma Chapman, Z. Mike Chirenje, Elizabeth Namukwaya, Simon Pini, Kennedy Nkhoma, Matthew J. Allsop

**Affiliations:** ^1^ Clinical Trials Research Centre University of Zimbabwe Harare Zimbabwe; ^2^ Academic Unit of Palliative Care, Leeds Institute of Health Sciences University of Leeds Leeds UK; ^3^ Department of Internal Medicine Makerere University Kampala Uganda; ^4^ Florence Nightingale Faculty of Nursing Midwifery and Palliative Care Cicely Saunders Institute, King's College London London UK

**Keywords:** access, evaluation, health care quality, neoplasms, palliative care, qualitative research, Zimbabwe

## Abstract

**Objective:**

Cancer patients in Zimbabwe typically access health services with advanced disease, limiting treatment choices and lessening the likelihood of positive treatment outcomes. We outline experiences of patients with advanced cancer prior to interaction with palliative care services to identify targets for future intervention development to enhance care delivery in Zimbabwe.

**Methods:**

Participants were purposively sampled adult patients with advanced cancer. We adopted a thematic approach to guide a qualitative secondary data analysis exploring factors influencing support sought by participants, external factors influencing decision making across the disease trajectory and the process for seeking and accessing palliative care.

**Results:**

Participants reported fragmented and uncoordinated care, from initial symptom experience and throughout disease progression. A recurring notion of disjuncture was present through participants' experiences of gaps, breaks and discontinuity across the disease trajectory. Each step had a beginning and end without clear routes for transition with movement between steps as a result of happenstance or informal encounters.

**Conclusion:**

Targets for intervention development at the patient and family level exist that may reduce the disjuncture currently experienced between need and care provision. A holistic response that incorporates engagement with policy actors is critical to addressing prominent financial constraints for patients.

## 1 INTRODUCTION

Cancer is the second leading cause of death globally (Are et al., 2018), causing 8 million deaths annually, with incidence and mortality projected to rise (Atun et al., 2015; Potts et al., 2018). It is anticipated that the disproportionate burden of cancer will worsen in low‐ and middle‐income countries (LMICs) where more than half of cancer deaths currently occur, yet cancer control efforts are given low priority (Fatunmbi et al., 2018). There is a large disparity in access to cancer treatment and outcomes worldwide and less developed countries account for only 6% of the total resources that are spent on cancer care. Such limited resources lead to limits in the availability of treatment and contribute to the high mortality rates in less developed countries (Swanson et al., 2018). In sub‐Saharan Africa, 70%–80% of the cancer cases are at a late stage and incurable at the time they are detected and diagnosed due to lack of access to cancer screening and diagnostic technologies, poverty and limited health literacy (Brien et al., 2013; Okunade et al., 2019; Shah et al., 2019). It is estimated that cancer incidence in sub‐Saharan Africa (SSA) will exceed 1 million in 2030 (Brien et al., 2013). Cancer is projected to be the lead cause of SSA experiencing the highest proportional increase in serious health‐related suffering globally by 2060 (Sleeman et al., 2019).

In Zimbabwe, cancer is a major cause of morbidity and mortality, with the most recent data available through the World Health Organization (WHO) indicating there were 16,083 new cancer cases and 10,676 deaths in 2020 (International Agency for Research on Cancer, 2021). The most common cancers are cervical and breast, accounting for a third of all cancer cases and predominantly affecting women, with prostate, Kaposi sarcoma and Non‐Hodgkin's lymphoma cancers also common. Despite a population‐based registry being present in Zimbabwe (Tangka et al., 2019), existing cancer data are thought to underestimate the extent of the cancer burden due to a lack of appropriate diagnosis, poor access to care and limitations in infrastructure (Olaleye & Ekrikpo, 2017). Furthermore, there is currently a lack of availability of data on the number of people living with cancer presenting at an advanced stage of disease in Zimbabwe. Regional estimates place these at 70%–80% of all patients with cancer prior to the coronavirus disease 2019 (COVID‐19) (Brien et al., 2013; Shah et al., 2019). However, it is anticipated that health system closures (e.g., suspending cancer screening programmes) and reductions in the availability of and access to care may lead to an initial reduction in cancer incidence which will be followed by an increase in advanced‐stage diagnoses and cancer mortality (Sung et al., 2021).

Broadly, across the research literature, patients with cancer and their caregivers report a wide range of context‐bound unmet needs (Wang et al., 2018). Patients with cancer in SSA have a high burden of physical symptoms, including lack of energy, shortness of breath, cough, pain, difficulties in sleeping and dry mouth. Patients also report wanting better communication around their needs and care options and experience psychological and spiritual distress (Adejoh et al., 2021; Beynon et al., 2014; Harding et al., 2011; Selman et al., 2011) alongside emotional grief owing to cancer stigma and perceptions of patients with cancer as being worthless (Edwards & Greeff, 2018). Living with cancer can have a pervasive impact on a patient's life including social relationships and spiritual and religious beliefs and practices (Kelly et al., 2019). The impact of patients extends to family caregivers too, who face compounded poverty and psychological distress, where the role can be viewed as a burden and challenging (Adejoh et al., 2021). Patients with cancer and their families are also faced with common and high out‐of‐pocket expenses across all stages of the disease trajectory, leading to decreases in total household income, reduced expenditure for social and recreational expenditure, the sale of family assets and the need to borrow money (Owenga & Nyambedha, 2018). Public health spending per capita in Zimbabwe is one of the lowest among countries in the Southern Africa sub‐region, with a lack of financial and social support systems by the central government (World Bank, 2017).

There is currently a limited evidence base underpinning cancer care in SSA, comprising mostly small‐scale quantitative studies to inform generic needs and outcomes of patients with cancer. Furthermore, there is a lack of qualitative evidence that outlines the specific needs and experiences of patients with cancer in SSA. The latter is a critical gap in the evidence base that is necessary to guide the development of patient‐centred approaches to health service delivery (Mirzaei et al., 2013) which we focus on with this study. Furthermore, the assessment of experiences, preferences, needs and outcomes of patients with advanced diseases including cancer has been highlighted as a regional priority in SSA (Harding et al., 2013; Powell et al., 2014). For those living with advanced disease, the WHO advocates for access to palliative care that provides ‘… an approach that improves the quality of life of patients (adults and children) and their families who are facing problems associated with a life‐threatening illness. It prevents and relieves suffering through the early identification, correct assessment and treatment of pain and other problems, whether physical, psychosocial or spiritual’ (World Health Organization, 2021). This research addresses gaps in the evidence base underpinning cancer care in SSA through undertaking a secondary qualitative data analysis to explore the experience of patients with cancer from the point of initially experiencing symptoms, through to access to palliative care services. By characterising the pathway of care and factors influencing access to support, we aim to identify targets for future intervention development to enhance the delivery of cancer care in Zimbabwe.

## 2 METHODS

### 2.1 Research question

What is the experience of people living with cancer before interaction with palliative care services in Zimbabwe?

#### 2.1.1 Study design and setting

Secondary data analysis (SDA) was carried out on a subset of data from a cross‐sectional, multi‐country qualitative study in sub‐Saharan Africa. SDA is a valuable and flexible method that can be used to explore new or supplementary research questions (Johnston, 2014). After primary analysis of the patient interviews conducted in Zimbabwe, varied and rich data on patient illness trajectories remained, and thus, we sought to further explore this data through SDA. We assessed the patient transcripts and the research question explored in this study against a rubric (Sherif, 2018) to assure the fit and relevance of pre‐existing qualitative data to this secondary analysis. Members of the research team were involved in the parent study, with the lead author responsible for undertaking interviews in Zimbabwe and the last author led the parent study and primary analysis with other members of the team. The parent study used semi‐structured face‐to‐face interviews with patients, caregivers, health professionals and policymakers to define optimal mechanisms through which patient‐level data can be integrated into palliative cancer care delivery and improvement. A total of 195 in‐depth interviews were conducted across the four stakeholder groups (advanced cancer patients (*n* = 62), informal caregivers (*n* = 48), health care professionals (*n* = 59) and policy‐makers (*n* = 26)). For patient participants, interviews sought to explore their experience of living with cancer, current interaction with and access to palliative care services, and the potential role of technologies to facilitate interaction with health services and data collection. In terms of the current delivery of palliative care for participants in this study, there is increasing evidence that early provision of palliative care, at least 3–4 months before death, can improve patient quality of life and reduce burdensome treatments and financial costs (Jordan et al., 2020). Similar to other low‐resource settings, however, in the context of Zimbabwe, palliative care predominantly supports people living with advanced diseases at the end of life, is largely supported by non‐governmental organisations and is not currently integrated into routine care (Khumalo & Maasdorp, 2016).

#### 2.1.2 Sample

From the original parent study sample, we selected all patient participants' data from Zimbabwe, comprising 20 patients with advanced cancer. Inclusion criteria for the parent study were patients aged at least 18 years, with an awareness of their diagnosis of advanced cancer (defined as those with metastatic cancer where, if possible, determined through histological, cytological or radiological evidence) and receiving palliative care. A purposive sampling frame identified potential participants with variation according to sex, income, family size and support, employment, cancer type and duration of time since referral to palliative care. For the parent study, data were collected between February and August 2019 in Zimbabwe. Participants in Zimbabwe were recruited through palliative care teams at district hospitals, which were both central hospitals, and a private voluntary hospice organisation.

#### 2.1.3 Data collection

In the parent study clinical staff at recruiting facilities reviewed clinical records to identify potential participants, introduced the study and referred them to the research assistants if they were willing to participate. Patients who were considered ethically inappropriate by members of the clinical team, for example, where death was imminent, were not approached. Participating patients were only asked to participate in one face‐to‐face interview and provided with between 24 h and 1 week to decide whether they would like to participate, at home or at a clinic. Trained researchers with an in‐depth knowledge of methodologies conducted the original interviews in English, alongside a proportion incorporating Shona (*n* = 16) as part of data collection in Zimbabwe, although language switching was common during interviews. The language of the interviews was chosen by the participant.

#### 2.1.4 Data analysis

**FIGURE 1:**
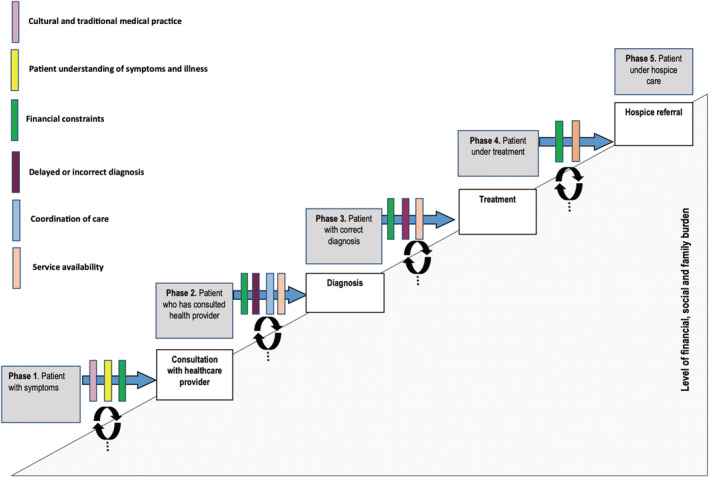
Phases of care accessed by patients and the barriers causing disjuncture between what is sought after and attainable

We adopted a thematic approach (Guest et al., 2012) to guide a qualitative secondary data as we sought to generate new theory underpinning the experience of patients living with cancer before interaction with palliative care services. Two researchers supported by a third author analyse transcripts one by one, with data coding comprising substantive and theoretical coding. The researchers read transcripts multiple times, paying attention to shared processes to determine meaningful components in the narratives that provided an understanding of how the participants experienced these events and produced an initial list of codes (Moran & Russo‐Netzer, 2015). When re‐reading the transcripts, we focused on the following questions: What are the factors that influence the type of support or treatment sought? How is the patient's decision‐making at each stage of the process affected by external aspects such as service costs? How do these factors affect the process of moving into palliative care services? Upon obtaining a sense of immersion in the material, the authors met to discuss initial findings. Strategies adopted included open and focused coding and memos, searching for negative cases and constant comparison, to develop categories and identify trajectory patterns from initial symptom experience to palliative care access. Theoretical coding of disjuncture emerged from constant comparison which was further explored through review of the research literature, including disjuncture theory (DePoy & Gilson, 2015; Jarvis, 2012). Disjuncture reflects a disconnected relationship between at least two entities, the patient and the healthcare they sought, often with a conflict between perceived need and attainment of support. Following analysis, we developed a schematic depicting principal themes and patterns in the analysis, partly informed by existing pathways of access to treatment (Scott et al., 2012). Members of the research team developed an initial thematic network which was then adapted through iterations of feedback to the wider research team. The final thematic network is presented in Figure 1. Data are presented in accordance with consolidated criteria for reporting qualitative research (COREQ) (Tong et al., 2007). Ethical approval was provided from local institutional review boards in Zimbabwe and collaborating organisations.

## 3 RESULTS

### 3.1 Findings

The clinical and sociodemographic characteristics of the participants are shown in Table 1. The average age of the participants was 51 years, ranging from 19 to 78 years, with multiple cancer types (see Table 1). Our coding framework included five stages of a patient's cancer trajectory from the initial appearance of symptoms to accessing hospice care, as illustrated in Figure 1. Themes comprised both exclusive sub‐themes leading to delays in accessing support, alongside a recurring concept of disjuncture (see Table 2). Supporting quotes are provided alongside the findings (see Table 3). Disjuncture in this context refers to the gaps, breaks or discontinuities in the patient experience of the cancer trajectory and the contingent factors that operate to account for or explain them (Bjursell, 2020). Within our analysis, these arose at the level of the patient, their family and professional health care and service. We outline the thematic network which includes the stages of the cancer trajectory, sub‐themes and the presence of disjuncture in Figure 1 and describe these in detail, below.

**TABLE 1:** Clinical and sociodemographic characteristics of patients (*n* = 20)

Demographic variable	Category	Number (%)
**Gender**	Male	10 (50)
	Female	10 (50)
**Marital status**	Married	10 (50)
	Unmarried	4 (20)
	Divorced	3 (15)
	Widow	2 (10)
	Widower	1 (5)
**Education**	None	1 (5)
	Primary	3 (15)
	Secondary	12 (60)
	College/university	4 (20)
**Age group (years)**	<30	4 (20)
	30–39	0 (0)
	40–49	4 (20)
	50–59	5 (25)
	>60	7 (35)
**Cancer type**	Anal	1
	Bladder	1
	Brain	1
	Breast	2
	Cervix	1
	Colon	1
	Leukaemia	2
	Lung	2
	Non‐Hodgkin's B cell lymphoma	2
	Ocular	2
	Penile	1
	Prostate	2
	Rectal	1
	Spinal	1
**Location of recruitment**	Private facility	12 (60)
	Central hospital	8 (40)
**Presence of histological confirmation of diagnosis**	Yes	19 (95)
	No	1 (5)
**Receipt of treatment**	Previously undergone surgery	7 (35)
	Current receipt of chemotherapy or radiotherapy	8 (40)

**TABLE 2:** Themes and subthemes of patient trajectory

Theme	Sub‐theme
Initial appearance and management of symptoms	Awareness and perceived seriousness of symptoms
	Cultural beliefs
	Financial constraints to seeking support for symptoms
Healthcare professional consultation	Moratorium through initial medicines management
	Financial constraints to diagnostic testing
	Delayed and incorrect diagnoses
Diagnosis	Availability of specialists and diagnostic testing
	Patient responsibility for coordination of care
Treatment	Limited options through delayed diagnosis
	Treatment as cost prohibitive
	Limited stock and facilities
Accessing hospice and advanced disease	Haphazard discovery of the hospice
	Increasing burden on family

**TABLE 3:** Quotes to support themes and sub‐themes derived from the analysis

Theme	Sub‐theme	Quote number	Supporting quote
**Initial appearance and management of symptoms**	***Awareness and perceived seriousness of symptoms***	1	‘*For me it has been a long way … since 2000 beyond 2000. I used to feel it on my organ [penis]‐; if l do some work, I would feel some pain on my organ [penis] … but I did not know what it was. After some time, the organ will be painless, not knowing that … its cancer’*. Male,75, Prostate cancer
	***Cultural beliefs***	2	*‘You know what happens in the rural areas that there are some who will be coming with with their own views. Some will say, “let us try this, there is this traditional medicine that can what‐; that can cure it,” and we would try that … The majority of people kept on saying “do the traditional procedures [consult traditional healers], do the traditional procedures, do the traditional procedures”, the rural community people’*. Male, 53, Ocular squamous cell carcinoma.
	***Financial constraints***	3	‘… *but the doctor I talked to said ah the issue that is there is that to just take your bone marrow while you are saying your financial situation is not okay… because I stay with my mom we are two and its me who was buying stuff and selling so as to help at home. My sister sends money here and there. So that is when they said, your financial situation does not allow us to take your bone marrow and you might not be eligible to have the Chemo [chemotherapy] … So, when I heard this I didn't know what to do then. I said ah doctor so you can wait on taking what, the bone marrow if it is expensive, then when I have gathered money I will come back and have what, the bone marrow process’*. Female, 27, Acute Leukaemia
**Health care provider consultation**	***Moratorium through initial medicines management***	4	*‘… we were told by this doctor … he is the one who realised that it was now a long time giving me ibuprofen‐; yes after a long time, he realised that I should … I should go to …<<name of central hospital>> … it was just pills ibuprofen only, ibuprofen only, ibuprofen only…you know. That is when the doctor surrendered and referred us to <<name of central hospital>>’*. Male, 75, Prostate cancer
		5	*‘Ahm when it started, I didn't know about what it was. I was going to a clinic and they were giving me treatment for piles but on noting that there was no change, they told me to go to <<name of central hospital>> where there are doctors. And even at <<central hospital>> they continued giving me the treatment for piles but I told them that it doesn't change. The pain is still the same.’* Female, 40, Anal squamous cell carcinoma
	***Financial constraints to diagnostic testing***	6	‘… *Then l went to the Family Planning Clinic. Then l was given appointment time; 9 months, I was asked to come back after 9 months with $90. Then l waited whilst my children were looking for the money that was required. When the money was raised, I went back on the date that I was booked’*. Female, 65, Cervical cancer
		7	*‘I … I have been ill for 7 years. I became ill in … 2012. “So, I needed help‐; I had no money, my husband is not employed. So, all along I failed to go to the hospital. So, I took a long time [before getting help]”’*. Female, 45, Breast cancer
	***Delayed and inaccurate diagnoses***	8	*‘Then I went to the local clinic, then I arrived there, then I was given‐; then they tested me. At first they said, “…there is nothing that has been observed.” I then went there for the second test, and they said,* “*… there are some lumps which have been noticed on what? On the liver.” “Then, they initially put me on TB [*i.e. *tuberculosis] treatment.” So, after observation, they said, “… giving you TB treatment is not the correct thing to do” … I then stopped taking those TB pills. Then I went back. And they said to me, “We are supposed to take [a piece] of your liver [*i.e. *perform a biopsy] … then they took a piece of my liver and then took it for examination … When I went back there for the next review, they said, “All our previous diagnoses about TB [*i.e. *tuberculosis] and so forth were wrong, It is what? It is cancer.” So, they told me that it was cancer. They then said, “… hold on. We shall tell you to go where? To a <<local central hospital>>.” They then gave me a letter (referral letter)”’*. Female, 25, Non‐Hodgkin's B Cell Lymphoma
		9	*‘So, when I went to <<name of mission hospital>> they administered various medications on me … they made me buy too many medications. I think there is no eye medication which I didn't purchase. Only until then‐; when they gave up on me. They said, we are now referring you so that you go to <<name of central hospital>>’*. Male, 54, Ocular squamous cell carcinoma
**Diagnosis**	***Availability of specialists and diagnostic testing***	10	*‘There were some instances where‐; when it [catheter] became so blocked that I had to go [to the hospital] very early in the morning … Then I went to the <<local central hospital>>…the doctors were on strike. and they refused to‐; to change it … Then I was lucky … I found student doctors … As I stood there, they just said, “You come here.” Then it was removed’*. Male, 50, Rectal tumour
		11	*‘… Then he told me, “Go to the laboratory in Baines [Avenue] … It is called <<name of laboratory>>.” Then I went to that place. When I went there, I paid some money … After paying the money, they told me, “The results will be out after, eh, 12 days.” I came back again after those 12 days’*. Male, 58, Penile cancer
	***Patient responsibility for coordination of care***	12	*‘Then they gave me those results. Then I said to them … “Look, I'm not able to comprehend what is written on your document.” They said … “go to <<name of central hospital>> again and see who? Your doctor.” When I came here [at the central hospital], I was then told, you are late. You should also have made an appointment with … with that doctor. Time was passing by. That growth was doing what? It was getting bigger. Then I made an appointment … When I handed the document [results] to him, he then told me, father, you have cancer on your organ [penis]. Then they actually gave me an appointment date and said, “You are supposed to come so that you can start treatment …”’*. Male, 58, Penile cancer
		13	*‘I would say … around February is when I started to become ill. I started visiting doctors in South Africa. Eh, I then came here, and then went to <<local referral hospital>> where they said they wanted what was coming from South Africa [referring to medical records] so that it enables them to know what to do. I think mother [referring to his mother] is making enquiries … so that he [the relative in neighbouring country] is able to find out where they [referring to clinician in South Africa] ended, so that those here [referring to local clinicians] know where to begin. Because they [referring to local clinicians] cannot proceed when they don't know what was discussed [by the clinicians] in South Africa‐;where they ended or where they begun, and where they left things’ Male, 60, Prostate cancer*
		14	*‘As it is, I'm puzzled about what the correct diagnosis is … We went there [to the hospice] and gave them the two X‐ray images which I had. They were taken by ‘xxx’. One was taken by ‘xxx’; the other was taken by staff at <<name of central hospital>>. So, the [doctor] at <<name of central hospital>> spoke his own views. ‘xxx’ was speaking what? His own views. They were now contradicting. ‘xxx’ [private doctor] is the one who said its cancer … We then went there [to central hospital] and he said, “No … we have not yet confirmed this disease … Let her undergo‐; let her return again … more X‐ray images be taken.” They took sputum as well. We want them to confirm what the condition is. That's all what is left so that we are then given the relevant medication …*’ Female, 52, Lung cancer
		15	*‘There is another one‐; I don't know where his surgery is located. He is the one who said go and see that one. We then went to … before we got to him, we saw this other one who has got this surgery … When we got there, he said you have come to the wrong person, l am not the one. “let me direct you to the person with this name, the one you are looking for.” Then he told him that “there are people who were referred to you by … by this doctor … he is the one who referred them to you”’*. Male, 75, Prostate cancer
**Treatment**	***Limited options through delayed diagnosis***	16	*‘Unfortunately, I was so weak for surgery … this is now cystectomy. Unfortunately, the‐the Urologist said I was too weak for that [sighs and takes a deep breath] he needed to give me time to go and rest.. “I started bleeding again, I came back …*. *but when I came back in January [2019] … I was referred for cystoscopy, [it] had spread now to the lymph nodes. And I was told by a Urologist that doing surgery at that juncture is a waste of time … This is when I was moved down here [to the adult oncology ward] for the‐; the palliative care”’*. Male, 71, Cancer of the bladder
		17	*And then I went, that was in <<my hometown>>… they started doing the‐; it's called staging, they wanted to see how far it had gone, and they … I went for some scans, CT scans, X‐rays, many‐; many tests. They told me that it has gone up to the stomach. So, they can't operate. At first, they told me that if its still on the early stage, they will put me a colostomy bag … they said they can't do that because it has spread up to the whole of the stomach, the intestines, there are a lot of cancer cells inside my stomach, so I will have to do chemotherapy*. Female, 40, Anal squamous cell carcinoma
	***Treatment as cost prohibitive***	18	*‘I came to see the <<name of central hospital>> team after I had been disappointed at <<name of mission hospital>>. Regarding this place [referring to central hospital], we were discouraged by the issue of payments; the fact that its expensive. So, we sought for missionary hospitals. However, that actually damaged me … my view is that, that is what damaged me … I used to hesitate on the issue of payments because the opportunity to get money eluded me’*. Male, 54, Ocular squamous cell carcinoma
		19	*‘The challenges that I'm talking about are that I had no money to undergo chemotherapy. After the operation they said I was supposed to go to << central hospital>> to do what? To undergo chemotherapy, and then Radiotherapy. So, that required money. I had no money. My husband passed away in 2012. So, the money I used to get was not enough to do what? To raise enough money to get treatment. So, I stayed on with my problem until it got to a situation which was unmanageable’. Female, 48, Breast cancer*
		20	*‘Because I might get to the point of surrendering and think that it is‐; it is better for me to die because I'm not able to do what? To afford the charges, but I would still be keen to live … I failed to raise money to cover the charges which they calculated for me. Then I retreated and withdrew. That disease did not stop. It continued to worsen’*. Male, 58, Penile cancer
	***Limited stock and facilities***	21	*‘I was operated on 9 October 2018. Then I was discharged on 24 October 2018, and I went to my rural home*. *So, after I had been discharged, they then said, I was supposed to undergo radiotherapy. I came time and again and found the machines down when I was supposed to undergo radiotherapy’* Male, 54, Ocular squamous cell carcinoma
**Accessing hospice and advanced disease**	***Haphazard discovery of the hospice***	22	*‘… I was told by my Aunt who used to go there she later died. She said, why don't you go the clinic so that you can be assisted. You can be assisted if its cancer. So, l went. I got there and l saw a Sister who was there … She said get checked for everything first, then after you are told that you have cancer then we will give you the medication …’* Female, 65, Cervical cancer
		23	*‘When I came to‐; to‐; to the hospice I had been referred by <<local clinic>> because I had become a person who was at a loss and I was unable to help myself. So, they saw it fit to send me to the hospice …’ Female, 48, Breast cancer*
	***Increasing burden on family***	24	*‘… I can say everything, everything will be a standstill go‐going backwards … for example right now I can say there is nothing I am capable of doing without, without taking my pills mmm so even with my wife it affects, now we have one, one year and half but we don't have a child. We don't have a child not because we are trying and failing but I am not … I am not producing, if we have sex I don't produce, so I will be saying, I will be blaming myself saying I am the one causing everything not to happen. So I would be thinking that cancer causes this, if I was okay I would do everything’*. Male, 19, Brain tumour
		25	*‘… it affects me when the lumps grow big. You will not be able to breathe and you lose strength. You would suffer from inexplicable illnesses. Take for instance the current situation; since I fell ill, I have not been able to kneel for a long time or to carry heavy objects. Concerning school, it actually affected me because I failed to write [examinations]. I failed to go back to school to learn. Up to this day, it still affects me’*. Female, 19, Non‐Hodgkin's T Cell Lymphoma
		26	*‘… I was affected … I was very affected. I ‐; currently I'm always like this, bed ridden. Seated on the blankets, on the bed. There is nothing I'm able to do … Also, this condition of illness has affected me in my work in particular. I used to be the person who worked in this home, working for the family. So … my children are suffering a lot due to my bedridden state in that I'm not working due to‐; there is nobody who is properly working … My children are sometimes sent away from school. I'm suffering as I'm seated here. When they get sent away from school, I get distressed and think, “Oh, they have been sent away from school. What do I do now?” Aah, this illness is distressing me’*. Female, 45, Breast cancer
		27	*‘… xxx “will go to the hospice,” my daughter: “She will go to the hospital.” “If there are some things that I need, that's where she gets them or the information about what we require …”’* Female, 45, Breast cancer

#### 3.1.1 Initial appearance and management of symptoms

##### Awareness and perceived seriousness of symptoms

Knowledge and perceptions about the seriousness of symptoms influenced patients' initial help‐seeking behaviours and time to cancer care. Most experienced signs of feeling unwell and symptoms including pain, bleeding, severe cough, shortness of breath and appearance of growths. Patients were not aware of the cause of symptoms and did not perceive them to be serious, often not initially seeking help. Some participants obtained medication for pain management from healthcare providers but most experienced a prolonged period of self‐management of early symptoms without input or support from healthcare providers (*quote 1*).

##### Cultural beliefs

Families and participant communities shaped interaction with health services when experiencing initial symptoms. Some families and communities interpreted symptoms as emanating from traditional or cultural causes and hence needed to be addressed with traditional medicine. Some participants described their families in rural areas suggesting and facilitating access to practitioners to manage their condition (*quote 2*).

##### Financial constraints

Participant decision‐making regarding engaging with care providers during early and subsequent stages is shaped largely by associated service costs, including transport, consultation fees, laboratory specimen fees and treatment. This was a major determining factor on whether a patient would move to the next level of care and central to the decision to approach a provider to determine the cause or treatment for the ‘symptom’. Almost all patients were aware that diagnosis and treatment required financial resources which involved drawing on support from family, relatives or friends before engaging health care providers (*quote 3*).

#### 3.1.2 Health care provider consultation

##### Moratorium through initial medicines management

When participants sought support with worsening or persistent symptoms there was no clear pathway alongside uncertainty in where to access support. Those who had previously visited healthcare providers revisited facilities. Access to and engagement with healthcare providers did not progress efforts to determine a diagnosis through testing, with durations of years reported between initial symptoms and receiving treatment (*quote 4*). Participants also reported periods of prolonged medication use despite an absence of improvement (*quote 5*).

##### Financial constraints to diagnostic testing

Persisting through interaction with health providers, the length of time between seeing the doctor and undergoing diagnostic tests, or an affordable selection, was affected by the ability of participants to pay. Family and friends were approached for support to gather the funds necessary to pay for diagnostic tests (*quote 6)*. Where funds could not be gathered by participants, long delays could be incurred (*quote 7*).

##### Delayed and inaccurate diagnoses

Inaccurate diagnoses were experienced by a quarter of participants across providers at primary health facilities and general hospitals, attributing initial cancer symptoms to conditions including tuberculosis and sexually transmitted diseases (*quote 8*). Participants again recalled narratives of management by a provider for a considerable time despite no improvement (*quote 9*). During delays in receiving a diagnosis, or recognition of their potential to indicate cancer, symptoms were progressing. Worsened symptoms without diagnosis or management led to some patients attending central care facilities in an effort to access support with its management.

#### 3.1.3 Diagnosis

##### Availability of specialists and diagnostic testing

Most participants attended central care hospitals to receive a diagnosis, prompted by inadequate resolution of symptoms with an existing provider or through referral. Most participants were able to access a specialist via this route, although a few participants reported labour strike action by doctors hindered access (*quote 10*). Limited diagnostic resources, including nonfunctioning equipment, at the central care facilities resulted in patients being referred to private facilities for further investigations which included laboratory tests and medical X‐rays, leading to participant travel between facilities to undergo tests and determine the appropriate treatment approach (*quote 11*).

##### Patient responsibility for coordination of care

Navigating which professionals and facilities should be accessed across the connecting and moving network was felt to be the responsibility of the participants, without knowledge of available resources to guide this process. At higher‐level facilities, such as tertiary hospitals, participants reported a lack of communication and poor coordination (e.g., interpreting diagnostic procedure results [*quote 12*]). Others reported having to undergo repeated tests when seeing a new doctor, leading to further delays in accessing biopsy services and pathology results. A few participants also underwent investigations in other countries such as South Africa, delaying receipt of medical reports to inform treatment plans (*quote 13*). One participant described disagreement between health providers as causing a delay in commencing treatment (*quote 14*). No referral system was described by participants, who were often moving between health care facilities driven by individual‐level decisions by health providers (*quote 15*).

#### 3.1.4 Treatment

##### Limited options through delayed diagnosis

As a consequence of the stage of disease at presentation, often for those who had incurred delayed diagnoses, limited treatment options were available (*quote 16*). A few participants reported being too frail to undergo the recommended treatment, with one participant no longer eligible for surgery because their cancer had metastasised (*quote 17*).

##### Treatment as cost‐prohibitive

For many participants, treatment was delayed due to the inability to pay. Some participants had commenced chemotherapy, radiotherapy or surgery (*quote 18*), funded through a variety of external sources (*quote 19*). Participants unable to raise the required finances expressed a sentiment of acceptance, hopelessness and fatalism about their situation, with some acknowledging a need to surrender (*quote 20*).

##### Limited stock and facilities

Following diagnosis and identification of appropriate management, access to treatments was limited by their availability. Chemotherapy drugs were not readily available at central care facilities and some patients had to purchase their own from private pharmacies or import them from other countries. Issues with radiotherapy machines were reported, inhibiting the completion of recommended treatment regimens (*quote 21*).

#### 3.1.5 Accessing hospice and advanced disease

##### Haphazard discovery of the hospice

Discovering and accessing the hospice was haphazard for most participants; there was no clear pathway or referral process reported by participants. Around half were made aware of the hospice through family and friends (*quote 22*), with others informed by healthcare providers (*quote 23*).

##### Increasing burden on family

The majority of participants described living with advanced illness as being a burden on their families. Most participants were the primary source of income for their household and had to leave formal or informal employment due to cancer‐related illness. The impact of the disease led to the breakdown of marriages, prevented plans to start a family (*quote 24*) and forced participants to leave education (*quote 25*).

A few participants reported that those caring for them had to forego employment and schooling to take care of them. For some participants, moving in with caregivers was their only option which participants felt placed an additional burden on them (*quote 26*). The family played an important role in providing emotional and tangible support for patients with advanced disease, accompanying participants to health care facilities for appointments and treatment, alongside collecting medication on their behalf (*quote 27*).

## 4 DISCUSSION

We describe the fragmented and uncoordinated experiences of patients with advanced cancer in Zimbabwe as they navigated support from early symptoms to hospice care. Across each stage of the cancer trajectory, multiple factors were highlighted that influenced access and engagement with health services and support. A recurring notion of disjuncture was noted through participants' experiences of gaps, breaks and discontinuity across each stage of the pathway: from accessing medical assistance about initial symptoms, obtaining a diagnosis, formulation and delivery of a treatment plan and accessing palliative care. Each step had a beginning and end without clear routes for transition, and an absence of mechanisms to effect continuity, such as formal referral processes from curative to palliative and hospice care. For most patients, movement between stages was the result of happenstance or informal encounters with someone known to the patient or a family member.

Timely recognition of cancer signs and symptoms is critical for early diagnosis and treatment initiation (Pati et al., 2013). The low awareness of presenting signs and symptoms has been highlighted as a major contributing factor to delay in accessing treatment, such as for women with breast cancer in SSA (Akuoko et al., 2017). Psychosocial factors which include beliefs held about medical and health practices have been shown as contributing factors to delayed cancer diagnosis and care (Brown et al., 2018). Approaches with emerging evidence of effectiveness at improving knowledge of cancer symptoms in the general population of LMICs include lecture and workshop series' (Mena et al., 2014) and computer‐based educational interventions (McCree‐Hale et al., 2012). There is limited evidence for any long‐term outcomes relating to disease downstaging and improved referral rates for media campaigns that target, for example, newspapers and national radio broadcasts (Qu et al., 2020). There is currently no literature reporting exploration of these approaches in Zimbabwe which may be a focus on future research. Furthermore, in our study, family members were a crucial component influencing initial interaction and access to support, whether through Western medicine, traditional medicine, or both simultaneously. The widespread availability and comparatively low cost of treatments from traditional practitioners can make these viable options for those seeking healthcare in Zimbabwe, as reflected broadly across research in the SSA region (James et al., 2018). At the health service level, some participants reported delays in accessing further support from primary health facilities due to a lack of symptoms being recognised as indicative of cancer. It was common for patients to report inaccurate interpretation of symptoms, including diagnoses such as tuberculosis or sexually transmitted diseases, aligned to evidence from Malawi and South Africa in which low knowledge of cancer signs and symptoms led to instances of misdiagnosis (Brown et al., 2018). The experience of participants in this study conveys factors at both the patient‐ and service‐level that may be targeted for future intervention development to improve outcomes for people with cancer. For example, one intervention approach that could address fragmented care is navigation services, where a person (navigator) engages with a patient to determine barriers to care and provides information to improve access to components of the health system, not just primary care (Peart et al., 2018). In the context of LMICs, there is evidence that the approach can improve screening rates, post‐operative complications and patient retention (Dalton et al., 2019). Broadly, there was a critical and significant lack of information for participants. Limited research from South Africa and Uganda has highlighted unmet information needs of patients with progressive illnesses, which include information on the causes and progression of diseases, symptoms, and financial/social support (Selman et al., 2009). It is crucial that to empower patients, approaches to improving the provision of information, independent of whether the mode is via navigation services or other modes of delivery, should be prioritised in future research. This extends to health professionals where, for example, limited or lack of cervical cancer knowledge among nurses especially in primary health care can act as a barrier to accessing prevention, screening and palliative care for women with cervical cancer in Zimbabwe (Tapera & Nyakabau, 2020). Targeting approaches to education across primary care is crucial, particularly in rural areas where 67.8% (World Bank, 2022) of the population resides. This may need to occur alongside existing efforts to extend access to people living with advanced cancer in rural areas, such as the Zimbabwe Rural Palliative Care Initiative (PCI‐Z) which sought to integrate palliative care in rural communities by expanding the services of existing home‐based care teams (Di Sorbo et al., 2010).

Alongside a focus on patient and service‐level interventions, there is a need to focus on the organisation of the health system itself in Zimbabwe, the structure of which leads to barriers to timely access to cancer treatments and care (Tapera et al., 2019). Efforts to support better coordination of services will require simultaneous engagement with policymakers. There is a supportive policy environment in Zimbabwe to foster development in the provision of care for cancer, with the most recent National Cancer Prevention and Control Strategy for Zimbabwe (Ministry of & Child Care, 2014) highlighting the need to map, coordinate and integrate what already exists, providing a clear steer for future research activity. An effective response to cancer requires strengthening all health system functions which includes service provision, delivery and resource generation across all stages of the cancer continuum (Knaul et al., 2015). For the majority of people with cancer presenting with advanced disease, existing challenges undermine efforts to increase the availability of palliative care. This includes a lack of access to essential medicines for symptom management due to, for example, factors relating to policy, education and training (Tererai, 2020), alongside an absence of chemotherapeutic drugs and radiotherapy (Das, 2021). These factors were highlighted in a recent project seeking to integrate palliative care into the Zimbabwe healthcare system (Sisimayi et al., 2021). Health institutions may have services with the potential to support palliative care delivery, but they lack trained palliative care personnel and experience persistent stock‐outs of essential palliative care medicines that can hamper optimal service provision. Any response to support the needs of people with advanced cancer must be holistic. Alongside wider health system development, it is also critical to include financing, which was the most notable and pervasive constraint for participants, often drawing on wider family and friends for financial support. In the context of LMICs, cancer can lead to an illness‐impoverishment cycle, with out‐of‐pocket spending wasted because it contributes nothing to improved health (Knaul et al., 2015) affected by often late detection limiting treatment options and effectiveness, purchasing of low‐quality or inappropriate care, and long waits to access care.

### 4.1 Strengths and limitations of the study

To our knowledge, this is the first study to explore patient experiences of living with cancer prior to interaction with palliative care services in Zimbabwe. Our purposive sampling frame was achieved in the parent study, ensuring perspectives from male and female participants, alongside different ages and cancer types, using broad, open‐ended questions about the experiences of participants seeking support and living with cancer. Secondary analysis was conducted across a multi‐disciplinary team, including the interviewer and research coordinators from the parent study. Limitations of the study include that the parent study had a different primary aim, meaning further elaboration and theoretical sampling to address the focus of this secondary analysis was not possible. Instead, we cycled backwards and forward through the data set to identify empirical accounts that could further deepen insights into, for example, the theoretical construct of disjuncture, and retained dialogue with existing concepts and literature sources throughout the analysis of transcripts. Our analysis also included a sample of participants living with advanced cancer who had accessed palliative care providers in Zimbabwe. The perspectives presented will not reflect the full range of experiences of those living with cancer and seeking support across the country. There will be diverse and alternative experiences of people living with cancer not reflected in this manuscript, but we provide important insights into the experiences of those who navigated the existing health system and accessed existing palliative care in the country.

## 5 CONCLUSION

The experience of a patient with cancer in Zimbabwe can be characterised by disjuncture, with unclear care pathways and an absence of mechanisms to effect continuity. This leads to delays in accessing care at each stage, from diagnosis to palliative care. Understanding the experiences of cancer patients and how they seek treatment and experience the health care system is vital in identifying opportunities for intervention development at the patient, service and policymaker levels. This qualitative study is the first of its kind to explore the navigation pathways of cancer patients in Zimbabwe, including cultural and familial factors, financial constraints, delays and uncoordinated care. This provides a basis to guide novel, context‐appropriate intervention development to improve access to and provision of care for people living with cancer and their families in Zimbabwe.

## CONFLICT OF INTEREST

None declared.

## Data Availability

The data that support the findings of this study are available on request from the corresponding author. The data are not publicly available due to ethical restrictions.
